# Therapeutic efficacy of lascufloxacin in patients with *Mycoplasma pneumoniae* pneumonia

**DOI:** 10.1128/spectrum.00255-25

**Published:** 2025-06-18

**Authors:** Naoyuki Miyashita, Makoto Ogata, Naoki Fukuda, Akihisa Yamura, Tomoki Ito

**Affiliations:** 1 First Department of Internal Medicine, Division of Respiratory Medicine, Infectious Disease and Allergology, Kansai Medical University 12880, Hirakata, Japan; LSU Health Shreveport, Shreveport, Louisiana, USA

**Keywords:** lascufloxacin, macrolide resistance, minocycline, *Mycoplasma pneumoniae*

## Abstract

The prevalence of macrolide-resistant *Mycoplasma pneumoniae* in Japan is increasing once again. This study compared the therapeutic efficacy of fluoroquinolone lascufloxacin with minocycline, the recommended first-choice drug for patients with macrolide-resistant *M. pneumoniae* pneumonia. A prospective observational study was conducted at 12 facilities affiliated with Kansai Medical University Hospital, investigating patients with *M. pneumoniae* pneumonia between January 2024 and January 2025. Of the 93 patients with *M. pneumoniae* pneumonia, 51 (54%) were found to be infected with macrolide-resistant *M. pneumoniae*. All of these patients had an A-to-G transition at position 2063 in domain V of the 23S rRNA gene, resulting in high-level resistance to macrolides. Of the 33 patients with macrolide-sensitive *M. pneumoniae* pneumonia, 91% and 90% of patients experienced defervescence within 48 hours of initiating antibiotics with lascufloxacin and minocycline, respectively. Among the 42 patients with macrolide-resistant *M. pneumoniae* pneumonia, 90% of those in the lascufloxacin group and 90% of those in the minocycline group experienced defervescence within 48 hours of starting antibiotics. No antibiotic changes were recorded for patients in either group. Our results demonstrate that lascufloxacin may be an effective treatment for macrolide-resistant *M. pneumoniae* pneumonia, even in highly resistant strains. Physicians may consider using lascufloxacin or minocycline instead of macrolides when macrolide-resistant *M. pneumoniae* pneumonia is suspected and defervescence does not occur within 48 hours of starting macrolide treatment.

**IMPORTANCE:**

Since the isolation of macrolide-resistant *Mycoplasma pneumoniae* in 2000, resistant strains have spread rapidly across East Asia. In Japan, the prevalence rate of macrolide-resistant *M. pneumoniae* has decreased since peaking in 2012. Nevertheless, 80–100% of *M. pneumoniae* strains in East Asia have become macrolide-resistant. Consequently, it was predicted that the number of macrolide-resistant strains would rise again in Japan. The Japanese Society of Mycoplasmology, therefore, recommended minocycline as the antibiotic of choice for treating macrolide-resistant *M. pneumoniae* pneumonia. Drug susceptibility to *M. pneumoniae* differs among respiratory quinolones, and the clinical efficacy of tosufloxacin, which has a high minimum inhibitory concentration, is inferior. Therefore, differences in efficacy may exist among respiratory quinolones. In this study, we investigated the therapeutic efficacy of lascufloxacin in patients with macrolide-resistant *M. pneumoniae* pneumonia, confirming that it was equally effective as minocycline.

## OBSERVATION

Lascufloxacin (Kyorin Pharmaceutical Co., Ltd.) is a novel fluoroquinolone antibacterial agent used to treat respiratory and otolaryngological infections (1). It exhibits potent antibacterial activity against major respiratory and otolaryngological pathogens, such as *Streptococcus pneumoniae*, *Haemophilus influenzae*, *Moraxella catarrhalis*, and *Mycoplasma pneumoniae*. It is also highly selective for bacterial targets, inhibits both DNA gyrase and topoisomerase IV, and exhibits minimal reduction in inhibitory activity in mutant strains (1, 2). A randomized, double-blind, comparative phase III study demonstrated that lascufloxacin treatment was highly effective from an early stage of administration and was well tolerated by patients with community-acquired pneumonia (CAP), including those with *M. pneumoniae* pneumonia (3).

The largest epidemic of *M. pneumoniae* infection ever recorded occurred in Japan in 2024. A key feature of this outbreak was the increased resistance of *M. pneumoniae* to macrolide antibiotics. Macrolides are significantly less effective clinically in patients with macrolide-resistant *M. pneumoniae* infections than in those with macrolide-sensitive infections (4–6). As there are no reports examining the effects of lascufloxacin in patients with macrolide-resistant *M. pneumoniae* pneumonia, we evaluated its therapeutic efficacy in such patients.

A prospective observational study was conducted at 12 facilities affiliated with Kansai Medical University Hospital, investigating patients who contracted *M. pneumoniae* pneumonia between January 2024 and January 2025. Physicians at the facilities collected nasopharyngeal swab specimens and, if available, sputum samples. Cultivation and polymerase chain reaction (PCR) testing for *M. pneumoniae* were carried out as previously reported (4–6). A direct sequencing method was used to search for mutations at sites 2063, 2064, and 2617 in the *M. pneumoniae* 23S rRNA domain V gene region in isolates or samples with a positive PCR result, as previously reported (4–6). The severity of pneumonia was evaluated using the A-DROP system of predictive rules proposed by the Japanese Respiratory Society (JRS) guidelines (7). Frequencies were compared using Fisher’s exact test. Between-group comparisons of normally distributed data were performed using a Student’s *t*-test. Skewed data were compared using the Mann–Whitney U test.

The therapeutic efficacy of lascufloxacin and minocycline against macrolide-sensitive and macrolide-resistant *M. pneumoniae* pneumonia was evaluated. The attending physicians made the antibiotic selection. All 18 patients admitted to the hospital were given lascufloxacin intravenously (300 mg on day 1, followed by 150 mg from day 2 onwards). Of these patients, four who were admitted to the intensive care unit were given lascufloxacin plus a steroid. The remaining 75 patients were given macrolides or quinolones orally. Lascufloxacin was administered at a dosage of 75 mg once daily and minocycline at 200 mg twice daily, in accordance with the package insert accompanying each drug. If clinical symptoms and signs had not improved by the second visit, the attending physicians changed the antibiotic treatment to minocycline or other quinolones.

We assessed the clinical outcome using fever, as previous studies have also only used the number of days with a fever to evaluate the clinical outcome in macrolide-resistant patients (4–6, 8–10). Additionally, we evaluated the therapeutic efficacy of antibiotic treatment after 48 hours, as previous reports have shown that the mean number of days with a fever in macrolide-sensitive patients during macrolide administration is between one and two days (4, 9, 10).

Of the 93 patients with *M. pneumoniae* pneumonia, 51 (54%) were found to be infected with macrolide-resistant *M. pneumoniae*. All of these patients had an A-to-G transition at position 2063 in domain V of the 23S rRNA gene (A2063G).

Table 1 presents the underlying conditions, pneumonia severity, laboratory data, and prognostic factors of patients with macrolide-resistant and macrolide-sensitive *M. pneumoniae* pneumonia. No significant differences were observed between patients with macrolide-resistant and macrolide-sensitive infections.

**TABLE 1:** Underlying conditions and clinical findings in patients with macrolide-sensitive and macrolide-resistant *Mycoplasma pneumoniae* pneumonia at the first examination^*a*^

Variables	Macrolide-sensitive *M. pneumoniae*	Macrolide-resistant *M. pneumoniae*	*P* value
No. of patients	42	51	
Median age (IQR), years	22.4 (16–48)	21.1 (16–42)	0.5176
No. of males/females	21/21	24/27	0.8365
No. (%) of patients with co-morbid illnesses	6 (14.3)	6 (11.8)	0.7636
No. (%) of patients with each pneumonia severity score^*b*^			
Mild to moderate	35 (83.3)	44 (86.3)	0.7748
Severe	5 (11.9)	5 (9.8)	0.7505
Extremely severe	2 (4.8)	2 (3.9)	>0.9999
No. (%) of patients hospitalized	9 (21.4)	9 (17.6)	0.7929
Laboratory findings, median (IQR)			
White blood cells, mean (µL)	6,150 (5,110–8,480)	6,500 (5,410–8,820)	0.2827
C-reactive protein, mean (mg/dL)	4.1 (1.3–9.1)	3.7 (1.2–9.0)	0.3174
Aspartate aminotransferase, U/L	28 (20–39)	26 (20–38)	0.4622
Alanine aminotransferase, U/L	25 (18–38)	24 (18–37)	0.6939
No. (%) of patients requiring invasive mechanical ventilation	2 (4.8)	1 (2.0)	0.5973
No. (%) of patients requiring ICU admission	2 (4.8)	2 (3.9)	>0.9999
No. (%) of deaths, <30 days	0	0	>0.9999

^*a*^ Continuous values are presented as medians and interquartile ranges and categorical/binary values as counts and percentages.

^*b*^ Patients were stratified into four severity classes by the A-DROP system: 0 point = mild, 1 or 2 points = moderate, 3 points = severe, and 4 or 5 points = extremely severe.

Table 2 shows the therapeutic efficacy of oral anti-*M*. *pneumoniae* antibiotics against both macrolide-sensitive and macrolide-resistant *M. pneumoniae*. All enrolled patients had a baseline fever of more than 38°C. Among patients with macrolide-sensitive *M. pneumoniae* pneumonia, lascufloxacin and minocycline demonstrated promising clinical outcomes, with fever resolution within 48 hours of antibiotic initiation observed in 91% and 90% of patients, respectively. Among patients with macrolide-resistant *M. pneumoniae* pneumonia, defervescence within 48 hours of starting antibiotics was observed in 90% of patients in both the lascufloxacin and minocycline groups. No antibiotic changes were recorded for patients in either group. Of the 14 patients admitted, intravenous lascufloxacin demonstrated good clinical efficacy, with defervescence observed within 48 hours of antibiotic initiation in all patients with macrolide-sensitive or macrolide-resistant *M. pneumoniae* pneumonia.

**TABLE 2:** Clinical efficacies of oral lascufloxacin and oral minocycline against mild to moderate macrolide-sensitive and macrolide-resistant *M. pneumoniae* pneumonia

	Macrolide-sensitive patients		Macrolide-resistant patients	
Variable	Lascufloxacin	Minocycline	Lascufloxacin	Minocycline
No. of patients	23	10	32	10
Mean age, years	22.8	22.0	21.8	20.6
No. of males/females	12/11	5/5	15/17	5/5
No. (%) of patients with defervescence within 48 hours after antibiotic administration	21 (91.3)	9 (90.0)	29 (90.6)	9 (90.0)
No. (%) of patients with antibiotic change at second visit	0	0	0	0

Since the first detection of macrolide-resistant *M. pneumoniae* in 2000, resistant strains have spread rapidly across East Asia (11–14). In Japan, the prevalence rate of macrolide-resistant *M. pneumoniae* has shown a downward trend since peaking in 2012 (15–18). This is thought to be mainly due to genetic replacement from the macrolide-resistant p1 gene type 1 to the macrolide-sensitive p1 gene type 2 lineage (15). A notable epidemiological feature of recent macrolide-resistant *M. pneumoniae* strains is the significantly higher macrolide resistance rate observed in type 1 lineage strains compared to type 2 strains. In the early 2010s, most type 1 clinical isolates were macrolide-resistant *M. pneumoniae*, whereas type 2 lineage strains were largely macrolide-sensitive *M. pneumoniae* (13, 15–17). Since the late 2010s, type 2 lineage strains have become clinically prevalent in Japan, and most of these are macrolide-sensitive *M. pneumoniae*. However, a gradual increase in the macrolide resistance rate of the type 2 lineage has been reported in China and South Korea, where 80–100% of *M. pneumoniae* infections are currently macrolide resistant (19, 20). As predicted, type 2 resistance has also increased in Japan.

Previous studies evaluating the efficacy of various antibiotics against macrolide-resistant *M. pneumoniae* pneumonia demonstrated that minocycline was the most effective (4–6). However, minocycline is contraindicated for children under eight years of age due to the risk of tooth discoloration and enamel hypoplasia. Therefore, the quinolone antibiotic tosufloxacin is recommended as an alternative treatment for children under eight years old with macrolide-resistant *M. pneumoniae* pneumonia. However, it is less effective than minocycline against macrolide-resistant *M. pneumoniae* pneumonia (4–6). Consequently, the Japanese Society of Mycoplasmology recommended minocycline as the preferred antibiotic for treating macrolide-resistant *M. pneumoniae* pneumonia (21). Drug susceptibility to *M. pneumoniae* differs among respiratory quinolones (22), and the clinical efficacy of tosufloxacin, which has a high minimum inhibitory concentration, is inferior (4–6). Therefore, there may be differences in efficacy among respiratory quinolones. In this study, we investigated the therapeutic efficacy of lascufloxacin in patients with macrolide-resistant *M. pneumoniae* pneumonia, confirming that it was equally effective as minocycline.

The JRS pneumonia guidelines recommend selecting antibiotics that are less likely to cause the development of resistance, primarily to prevent drug resistance. Lascufloxacin is an antibiotic with a mutant prevention concentration rate of over 100%, making it less likely to cause resistance. Therefore, the JRS guidelines recommend it as the first-choice drug for treating community-acquired pneumonia.

In the present study, there were too few *M. pneumoniae* isolates to perform multi-locus sequencing typing analysis. In future studies, it will be necessary to analyze mutations in multiple gene regions, classify *M. pneumoniae*, and evaluate the effectiveness of treatment based on classification.

In conclusion, our results demonstrated that lascufloxacin may be an effective treatment for macrolide-resistant *M. pneumoniae* pneumonia, including highly resistant strains. When macrolide-resistant *M. pneumoniae* pneumonia is suspected and there is a lack of defervescence within 48 hours of starting macrolide treatment, physicians might use lascufloxacin or minocycline instead of macrolides.

## Data Availability

The data presented in this study are available in the article.
